# Crosstalk between fibroblasts and T cells in immune networks

**DOI:** 10.3389/fimmu.2022.1103823

**Published:** 2023-01-09

**Authors:** Byunghyuk Lee, Seung-Hyo Lee, Kihyuk Shin

**Affiliations:** ^1^ Department of Dermatology, College of Medicine, Pusan National University, Busan, Republic of Korea; ^2^ Graduate School of Medical Science and Engineering, Korea Advanced Institute of Science and Technology, Daejeon, Republic of Korea; ^3^ R&D Division, GenoFocus Inc., Daejeon, Republic of Korea; ^4^ Department of Dermatology, Pusan National University Yangsan Hospital, Yangsan, Republic of Korea; ^5^ Research Institute for Convergence of Biomedical Science and Technology, Pusan National University Yangsan Hospital, Yangsan, Republic of Korea

**Keywords:** fibroblasts, T cells, cancer, autoimmune disease, immune response, inflammation

## Abstract

Fibroblasts are primarily considered as cells that support organ structures and are currently receiving attention for their roles in regulating immune responses in health and disease. Fibroblasts are assigned distinct phenotypes and functions in different organs owing to their diverse origins and functions. Their roles in the immune system are multifaceted, ranging from supporting homeostasis to inducing or suppressing inflammatory responses of immune cells. As a major component of immune cells, T cells are responsible for adaptive immune responses and are involved in the exacerbation or alleviation of various inflammatory diseases. In this review, we discuss the mechanisms by which fibroblasts regulate immune responses by interacting with T cells in host health and diseases, as well as their potential as advanced therapeutic targets.

## Introduction

As the principal components of connective tissues, fibroblasts are known to contribute to the formation and maintenance of extracellular matrix components and their remodeling, consequently playing pivotal roles in tissue development, differentiation, and repair. Therefore, their roles in immune responses have long been overlooked. Previous studies have identified the heterogeneity of fibroblasts based on their origins, surface markers, and functions using genetic lineage-tracing and single-cell RNA sequencing, and suggested the mechanism underlying the interaction of local immune cells in cancers and autoimmune disorders (1). At the site of inflammation, fibroblasts communicate with immune cells *via* secretion of cytokines and chemokines and direct cell-cell contact (2, 3). These interactions induce the recruitment of immune cells and produce inflammatory mediators; infiltrated immune cells play important roles in the phenotypes and functions of fibroblasts (1, 3). This suggests that fibroblasts have additional roles as members of immune networks while being a key structural compartment in different organs.

Fibroblasts play different roles in pathogenesis at different anatomical positions in diseases. They have diverse phenotypes with flexible responses to signals from microenvironmental niches. Disease-specific fibroblasts, such as cancer-associated fibroblasts (CAFs) and fibroblast-like synoviocytes in rheumatoid arthritis (RA), can directly interact with immune cells. T cells are important representatives of immune cells and are involved in the pathogenesis of cancers and autoimmune disorders. In addition, many studies have demonstrated that T cells participate in the deterioration or alleviation of diseases by interacting with tissue-specific fibroblasts.

Here, we discuss the complex crosstalk between fibroblasts and T cells in host health and disease. Interestingly, targeting fibroblast-T cell crosstalk could be a noteworthy therapeutic strategy for the treatment of diseases.

## Interaction of lymph node fibroblasts and T cells in host health and disease

Lymph nodes (LNs) are specialized organs wherein adaptive immune response is initiated and organize into distinctive compartments by highly specialized fibroblastic reticular cells (FRCs) (1, 4). FRCs contribute to the maintenance of immune homeostasis by supporting LN structures that offer distinct microenvironments and interact with immune cells by recruiting them and presenting antigens (5–7). Recent studies, such as single-cell analysis, high-resolution imaging, and various reporter mice studies, have identified the heterogeneity of FRCs. Depending on their position, phenotypes, and functions, FRC subtypes consist of marginal reticular cells, interfollicular FRCs, T-B border reticular cells, T-zone reticular cells (TRC), deep cortex periphery reticular cells, and medullary reticular cells. To participate in immune responses, FRCs produce cytokines, chemokines, and growth factors, thereby supporting the survival, activation, proliferation, and differentiation of immune cells. TRCs control T cell positioning, survival, and differentiation. TRCs directly regulate T effector functions *via* nitric oxide or constitutive cyclooxygenase enzymes (8–11). Co-culture of human FRCs with CD8+ or CD4+ T cells shows that FRCs can induce T cell anergy by producing indoleamine-2,3-dioxygenase, adenosine 2A receptor, prostaglandin E2, and transforming growth factor β (TGF-β) receptor, which limits T cell proliferation (12). In a co-culture of mouse FRCs with CD8+ T cells, FRCs induce an increased production of IL-2 and tumor necrosis factor (TNF) in CD8+ T cells (13). T cell survival, proliferation, and migration are affected by TRC-derived molecules such as IL-7, IL-15, IL-33, delta-like 4, CXCL12, CCL19, CCL21, and CD40 (5). T cells, which are regulated by FRCs, can influence the FRC phenotype and function. Activated CD8+ T cells can elicit TRCs to produce more immunostimulatory molecules, including the ICOS ligand, CD40, and IL-6 in a co-culture system (5, 13).

LN-FRCs have received much attention as key participants in tumor progression. In the colon adenocarcinoma model, LN-FRCs suppress an anti-tumor immune response, consequently contributing to the premetastatic microenvironment (14). In the B16 melanoma model, FRCs in tumor draining LN(dLNs) alter the secretion of ECM components, cytokines, and chemokines, and assist immune cell recruitment, activation, and differentiation, facilitating the establishment of immunosuppressive niches (15). In addition, TRCs in tumor dLNs restrain the expression of CCL21 and IL-7, thus limiting CD4+ T cell priming (15).

FRCs can support immune homeostasis and protect tissues from autoimmune responses (5). Homeostasis of T regulatory (Treg) cells, which have critical roles in balancing immune responses in mouse LNs, is maintained by MHC class II FRCs (16, 17). In addition, LN-TRCs elicit CD4+ T cell differentiation through the trans-presentation of CD25 to naïve CD4+ T cells. CD25-deficient LN-TRCs induce deterioration of autoimmune diseases by enhancing IL-17-producing T helper type 17 (Th17) cell differentiation (18).

IL-33 constitutively produced by FRCs can assist in the anti-viral CD8+ T cell responses in acute and chronic lymphocytic choriomeningitis virus (LCMV)-infected animal models (19, 20). In addition, FRCs present antigen to naïve LCMV-specific CD8+ and CD4+ T cells by MHCI and MHCII, respectively (6). In acute LCMV models, the absence of type I interferon-α receptor on FRC impaired the expansion of LCMV specific CD8+ T cells and the acquisition of effector phenotypes (21). Taken together, LN-FRCs play diverse roles in the immune system by controlling the homeostasis, proliferation, and function of T cells.

## Interaction of fibroblasts and T cells in cancer

**Figure 1:**
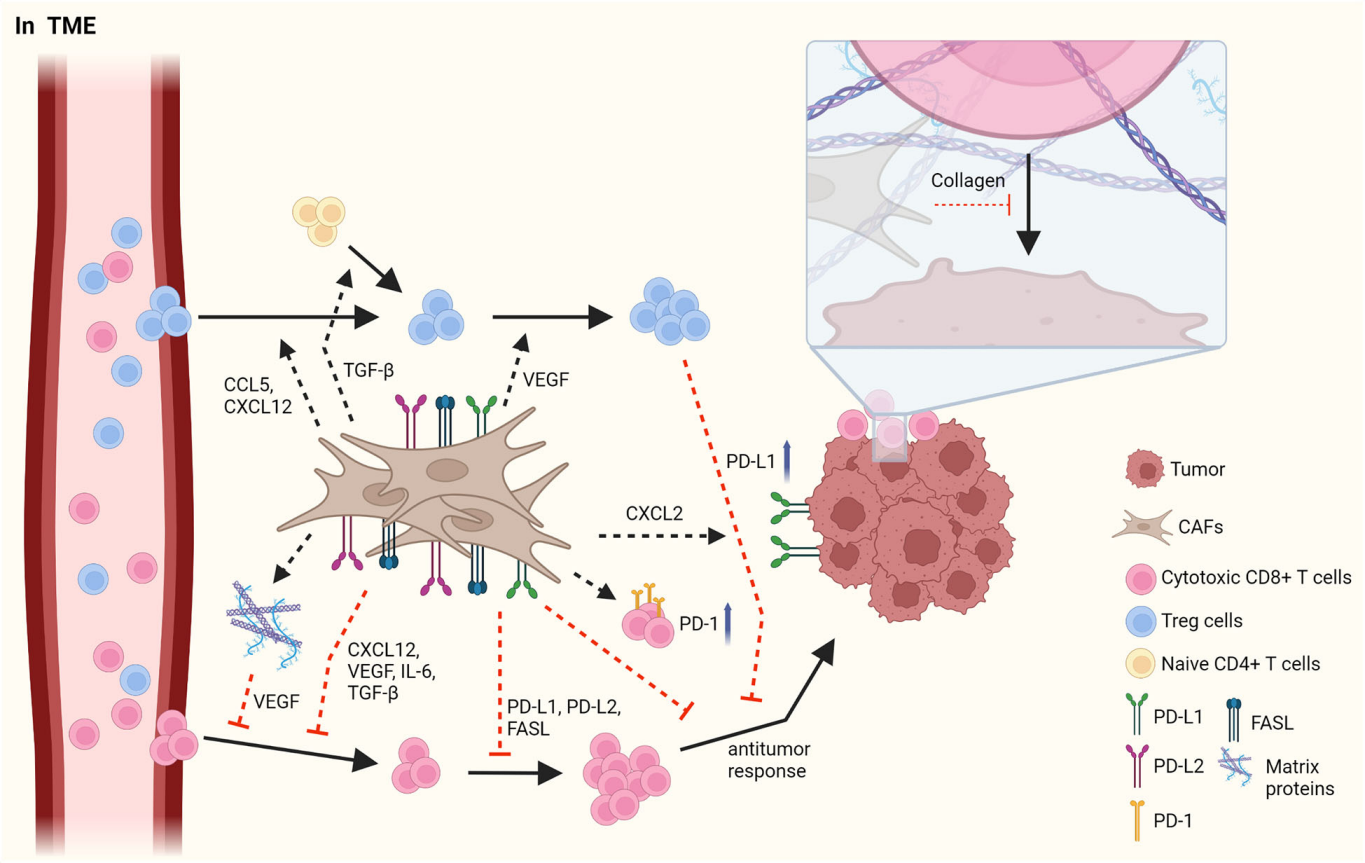
The interaction between CAFs and T cells in TME. CAFs have immunosuppressive roles by affecting Treg cells and cytotoxic CD8+ T cells. CAFs elicit the infiltration of Treg cells by secretion of CCL5 and CXCL12 and increase the proliferation of them through VEGF. In addition, CAFs facilitate the skewing of naïve CD4+ T cells to Treg cells. However, CAFs obstruct the migration of CD8+ T cells *via* various cytokines and chemokines such as CXCL12, VEGF, IL-6, and TGF-β. CAFs decrease the frequency and the activation of CD8+ T cells by PD-L1, PD-L2 and FASL. In addition, CAFs suppress an anti-tumor response of CD8+ T cells by upregulating PD-1 expression on the surface of T cells and by increasing PD-L1 expression on tumor through CXCL2. LRRC15+ CAFs induced by TGF-β signaling regulate anti-tumor response of CD8+ T cells. Furthermore, the accumulation of matrix protein, which is produced by CAFs can obstruct the migration of T cells and collagen can block the access of T cells to tumor, limiting the anti-tumor activity of T cells. Figure is generated with biorender (www.biorender.com).

The tumor microenvironment (TME) is mainly composed of tumor cells, immune cells, cancer-associated mesenchymal cells, and endothelial cells, and it plays an instrumental role in tumorigenesis and immune responses (1, 22, 23). CAFs, which are important mesenchymal cells in the TME, have heterogeneous cell phenotypes and functions. The roles of CAFs in TME have been widely described, and CAFs have been shown to facilitate tumor progression in most cancers through the production of cytokines, chemokines, and growth factors (**Figure 1**) (1, 22).

Distinct subsets of T cells, such as cytotoxic CD8+ T cells, Th cells, and Treg cells, play essential roles in regulating anti-tumor responses, and interactions between CAFs and T cells in the TME have been reported. Infiltrating Treg cells orchestrate immunosuppressive responses. CAFs facilitate the migration of Treg cells into the TME *via* CCL5 or CXCL12, thus increasing the frequency of these cells (24, 25). Infiltration of Treg cells is also enhanced by CCL17 and CCL22, which is induced by decreased expression of CD68 in CAFs and is also maintained by CAF-derived vascular endothelial growth factor-A (26–28). Moreover, Treg cells are differentiated from naïve T cells by inducing Foxp3 expression, which is facilitated by TGF-β from CAFs (29). Contrarily, the proliferation of CD4+Foxp3+ Treg cells has been reported to have improved due to myofibroblast exhaustion in pancreatic ductal adenocarcinoma (PDAC) (28). These results indicate that CAFs play an opposite role to Treg cells in the TME.

CD8+ T cells have anti-tumor responses by promoting the apoptosis of tumor cells. In contrast to their cytotoxic activities, CAFs suppress the infiltration, growth, and anti-tumor response of CD8+ T cells. Pancreatic stellate cells(PSCs), which can be altered into activated CAFs by TGF-β and platelet-derived growth factor (PDGF), reduces the infiltration of CD8+ T cells into tumor sites by attracting CD8+ T cells toward PSCs through CXCL12 expression (30). In response to hypoxia induced by CAF-mediated ECM modification, the angiogenic factor VEGF secreted by CAFs hinders the migration of CD8+ T cells into the TME (31, 32). In addition, CAFs induce the reduced CD8+ T cell infiltration by secreting IL-6 and TGF-β, leading to a decreased anti-tumor activity. Clinical trials on the blockade of IL-6 have shown enhanced T cell function and improved prognosis in patients (33, 34). In tumorigenesis of pancreatic adenocarcinoma, the formation of leucine-rich-repeat-containing protein 15(LRRC15)+ CAF is increased by TGF-β signaling (35). The depletion of LRRC15+ CAF induces the anti-tumor response of CD8+ T cells with increased expression of TNF and IFN-γ and also enhances the efficacy of anti-PD-L1 therapy (35).

When expressed on the surface of T cells and tumor cells, immune checkpoint molecules prevent the function of T cells. CAFs can disturb the anti-tumor activity of effector T cells by inducing the expression of immune checkpoint molecules, such as FAS/FASL and PD-1/PD-L1 or PD-L2, on their surface, followed by decreased CD8+ T cell frequency and activation and, in contrast, increased tumor cell viability (36, 37). CAFs are also able to increase immune checkpoint molecules in tumor and immune cells by secreting diverse cytokines (37, 38). CAFs upregulate PD-1, CTLA-4, TIM-3, and LAG-3 expression on both CD8+ and CD4+ T cells, resulting in hampered proliferation of T cells and impaired recognition of tumor cells in pancreatic cancer (39).

CAFs can inhibit T cell recruitment into the TME by having ECM proteins as a physical barrier, thus restricting their involvement in anti-tumor responses. A previous report indicated that the increase of collagen in neighboring tumor cells obstructs the interaction between T cells and tumor cells in lung and pancreatic cancers (40). Hypoxia-derived VEGF, caused by the accumulation of various matrix proteins in the ECM, can also decrease the migration of T cells into the TME (31, 32). Overall, CAFs promote the initiation and progression of cancer through several mechanisms, such as an increase in immunosuppressive responses of Treg cells and suppression of anti-tumor responses of CD8+ T cells.

## Interaction of fibroblasts and T cells in autoimmune diseases

### Rheumatoid arthritis

**Figure 2:**
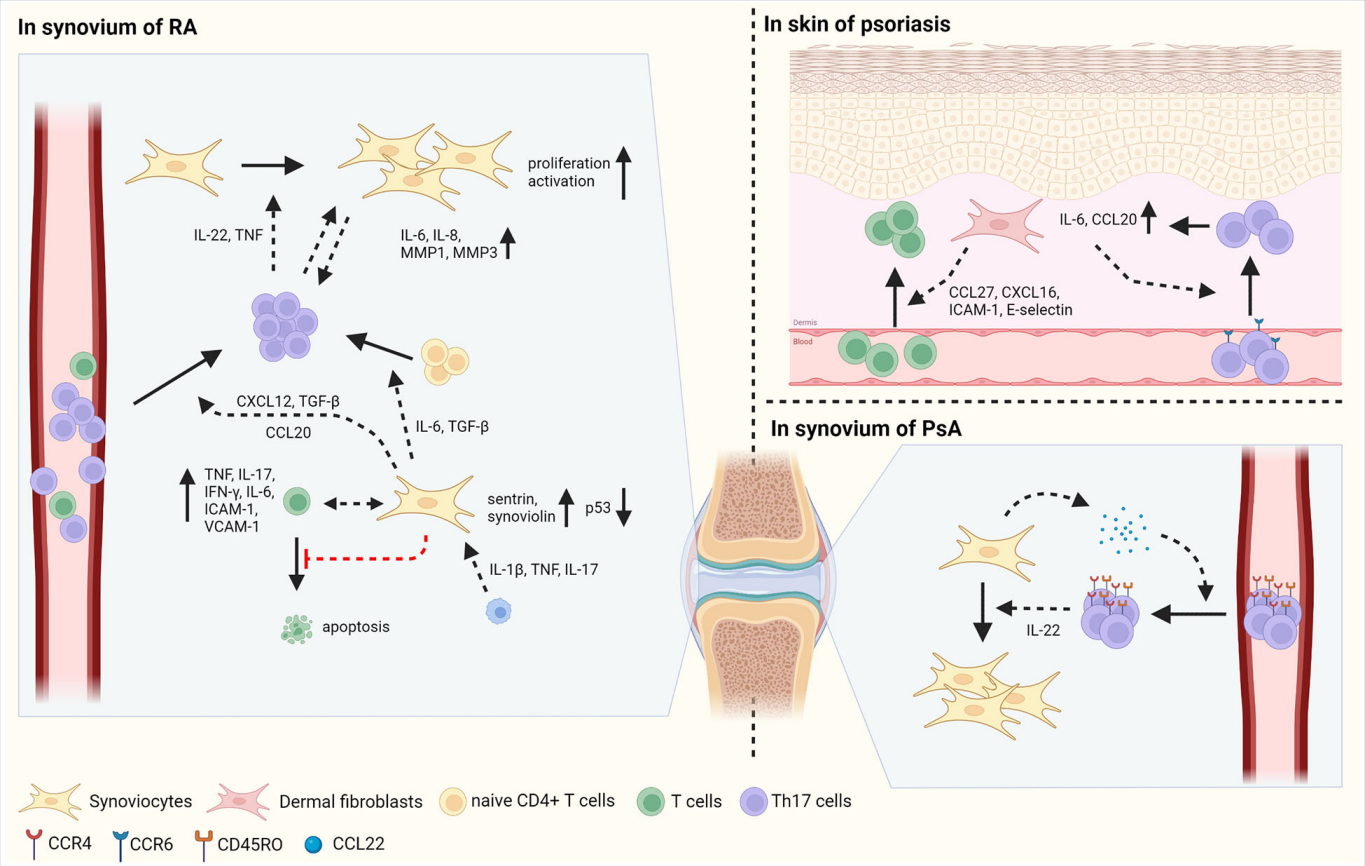
The interaction between fibroblasts and T cells in autoimmune diseases. Fibroblasts play inflammatory roles in autoimmune disorders such as RA, psoriasis, and PsA. In synovium of RA, synoviocytes have the mutation of p53, a tumor suppressor and increased expression of sentrin and synoviolin, which are anti-apoptotic proteins, inducing proliferation of synoviocytes. Synoviocytes elicit the differentiation of Th17 cells from naïve CD4+ T cells by IL-6 and TGF-β production. In addition, synoviocytes enhance the migration of Th17 cells *via* CXCL12 and TGF-β. IL-1β, TNF and IL-17 secreted by immune cells facilitate the production of CCL20 in synoviocytes, increasing the infiltration of Th17 cells into synovium. Infiltrated Th17 cells secret IL-22 and TNF, consequently improving the proliferation and activation of synoviocytes. Moreover, the contact between Th17 cells and synoviocytes induces the secretion of IL-6, IL-8, MMP-1, and MMP3 in synoviocytes. In cell-cell contact manner, synoviocytes increase the expression of TNF, IL-17, IFN-γ, IL-6, ICAM-1, and VCAM-1 in activated T cells. Besides, synoviocytes suppress the apoptosis of T cells by increasing survival signals and decreasing death signals. In psoriasis, fibroblasts produce CCL27, CXCL16, ICAM-1, and E-selectin, enabling to increased migration of T cells into skin. In addition, CCL20 and IL-6 produced by fibroblasts facilitate the infiltration of CCR6+ Th17 cells, followed by increased expression of IL-6 and CCL20 in fibroblasts. In PsA, synoviocytes elicit the migration of CD45RO+ Th17 cells by the secretion of CCL22. Then, infiltrated Th17 cells promote the proliferation of synoviocytes *via* IL-22 production. Figure is generated with biorender (www.biorender.com).

Synoviocytes, mesenchymal cells of the synovium, have distinct characteristics in RA compared with osteoarthritis (OA) and dermal fibroblasts in RA (**Figure 2**) (1, 41). Synoviocytes in RA show increased proliferation with defective expression of the tumor suppressor p53. Researchers have highlighted the mutations in the gene encoding p53 in synoviocytes of patients with RA (42). In addition, the expression of anti-apoptotic proteins, such as sentrin and synoviolin, is increased in the synoviocytes of patients with RA (43, 44). Synoviocytes consistently interact with infiltrating immune cells, especially T cells, thereby increasing the recruitment, differentiation, activation, and survival of T cells (45, 46). CD34- Thy1+ fibroblasts, which is highly expanded in RA patients, induce T cell infiltration into synovium, thereby increasing tissue inflammation (47). Increased migration of T cells to the synovium promotes interaction between T cells and synoviocytes, consequently contributing to disease chronicity (48). Infiltrating T cells are retained in the synovium by synoviocyte-derived CXCL12 and TGF-β (49, 50). Pro-inflammatory cytokines, such as IL-1β, TNF, and IL-17, produced by immune cells increase CCL20 secretion by synoviocytes, subsequently enabling CCR6+ Th17 cell recruitment (51–53).

Synoviocytes also regulate T cell differentiation *via* the production of diverse cytokines. For example, TGF-β and IL-6 produced by synoviocytes facilitate Th17 differentiation (54, 55). Synoviocytes from RA patients also contribute to the expansion of CD4+ T cells and increase the proportion of T cells expressing TNF, IFN-γ, and IL-17 in the synoviocyte-T cell co-culture system (56, 57). In addition, synoviocytes suppress T cell apoptosis by sustaining survival-related signals and restraining cell death signals (58, 59). T cells influence synoviocytes, increasing their proliferation, invasive capacity, and MMP secretion (57). In RA synovium, the production of IL-22, mainly by Th17 cells, is promoted and enhances the proliferation of synoviocytes. In addition, activated T cells can promote synoviocyte activation *via* TNF. Interactions between synoviocytes and activated T cells improve the production of TNF, IL-17, IFN-γ, IL-6, ICAM-1, and VCAM-1 by synoviocytes in a cell-contact-dependent manner (60). Th17 cells, but not Th1 cells, act as inducers of IL-6, IL-8, MMP1, and MMP3 production when co-cultured with synoviocytes of patients with RA, implying that Th17 cells are crucial subtypes in a pro-inflammatory feedback loop with synoviocytes (61). Thus, highly proliferative synoviocytes influence the function of T cells by expressing cytokines, chemokines, and cell adhesion molecules that promote inflammatory responses in RA.

### Psoriasis and psoriatic arthritis

Distinct types of cells are present in the skin, including keratinocytes and skin fibroblasts in the epidermis and dermis, respectively. Fibroblasts in psoriasis and PsA have similar characteristics (3); they have pro-inflammatory effects by interacting with T cells (**Figure 2**). Initiation of inflammation induces T cell migration into inflamed skin *via* CCL27 and CXCL16 and adhesion molecules, such as ICAM-1 and E-selectin (3). Fibroblasts present in psoriatic lesions produce higher levels of CCL27 than those in non-lesioned or healthy individuals. Moreover, in the lesions of patients with psoriasis, CCR10+ cells are more abundant than compared in healthy individuals, facilitating T cell-mediated inflammation *via* CCL27-CCR10 interaction (62).

Interactions between T cells and mesenchymal cells increase the production of IL-17, a pivotal cytokine in the pathogenesis of psoriasis and PsA (63). IL-17 activates skin mesenchymal cells, including fibroblasts and keratinocytes, and induces the production of the pro-inflammatory cytokine IL-6 and chemokine CCL20 in mesenchymal cells (64, 65). CCL20 elicits the recruitment of CCR6+ Th17 to the lesional skin, consequently contributing to the progression of psoriasis (65, 66).

In the synovium of patients with PsA, the levels of ICAM-1 and VCAM-1 expression and the number of T cells are similar to those observed in the synovium of patients with RA (67). CCR4 and CCL22, which are necessary for T cell infiltration into the skin, are abundant in the synovial fluid of patients with PsA (68, 69). Most CCR4+ CD4+ T cells in the synovium of patients with PsA also express CD45RO, a memory T cell marker, implying that the CCL22-CCR4 axis attracts memory T cells to the joint (69). Compared to patients with OA, patients with PsA have a higher concentration of IL-22 in the synovial fluid. T cells from the synovial fluid of patients with PsA produce large amounts of IL-22. *In vitro* studies have revealed that IL-22 facilitates increased proliferation of PsA synoviocytes, leading to deteriorated development of PsA (70). Further studies are required to compare the characteristics of PsA synoviocytes with those of RA synoviocytes, and their disease-specific interactions with infiltrated T cells. Overall, fibroblasts facilitate inflammatory responses by recruiting and activating Th17 cells through the secretion of cytokines and chemokines and increased expression of cell adhesion molecules in the skin of psoriasis patients and in the synovium of patients with PsA.

## Interaction of fibroblasts and T cells as a therapeutic target

As mentioned above, fibroblasts interacting with T cells participate in the pathogenesis of cancers and autoimmune disorders by producing cytokines and chemokines and promoting the survival and proliferation of T cells (1, 3). Therefore, targeting these interactions has received attention as a potential therapeutic option. Numerous approaches have been developed to date, such as direct targeting of cells and indirect targeting of the cytokine-receptor required for cell interaction (1, 3). To directly target disease-specific fibroblasts in different diseases, identification of the specific markers of fibroblasts in distinct disease-associated environments is required; however, it is difficult to determine selective markers for fibroblasts. As fibroblast activation protein (FAP) is one of the proteins that are highly expressed on CAFs in various cancers, FAP+ CAF is efficiently depleted by diverse approaches like immunotoxin, antibodies, DNA vaccines, and chimeric antigen receptor T cells, thereby contributing to the attenuation of tumor growth (71–73). The elimination of FAP+ CAFs increases T cell infiltration and enhances the efficacy of immune checkpoint inhibitors (74). Nevertheless, because FAP is also expressed in normal tissues, it can cause side effects, such as cachexia and anemia. In addition, depletion of α smooth muscle actin in CAFs facilitates undifferentiated tumor growth and subsequently decreases patient survival (28). Another approach to the direct killing of fibroblasts is the induction of apoptotic signals. To that end, delivery of PUMA, a pro-apoptotic gene, *via* viral vectors facilitates extensive cell death of synoviocytes *in vitro* and in the joints of rats with adjuvant-induced arthritis, resulting in decreased joint inflammation and destruction (75). Treatment of RA synoviocytes with cadmium enhances cell apoptosis and inhibits the production of pro-inflammatory cytokines. This effect was observed in rats with adjuvant-induced arthritis, whose clinical scores were alleviated, and joint destruction was protected (76).

An alternative strategy to indirectly target cell interactions has also been investigated. For instance, the CXCR4 inhibitor AMD3100 can restrict CXCL12-mediated T cell exclusion and promote T cell infiltration into the TME (77). In RA, psoriasis, and PsA, the infliximab (against TNF) reduces the infiltration of T cells into the synovium, and consequently decreases T cell-mesenchymal cell interactions (78–80). In addition, tocilizumab, a monoclonal antibody against the IL-6 receptor, diminishes the frequency of Th17 cells and production of cytokines secreted by Th17 cells in RA (81). Similar to RA treatment, tofacitinib, a JAK inhibitor, blocks the TNF-induced production of chemokines by synoviocytes and consequently limits immune cell infiltration. Peficitinib, another JAK inhibitor, decreases the production of pro-inflammatory mediators and inhibits the migration and proliferation of synoviocytes (82, 83).

## Conclusion

Recently, the involvement of fibroblasts in the regulation of immune responses by interacting with immune cells has been investigated. Although the heterogeneous and multifaceted characteristics of these cells have made it difficult to identify their roles in health and diseases, recent studies have elicited their potential to control immune networks using advanced technologies. Here, we have discussed the crosstalk between fibroblasts and T cells, the modulation of immune balance, and the pathogenesis of inflammatory diseases. Fibroblasts crucially participate in immune responses by interacting with T cells in secondary lymphoid organs and disease sites. Owing to their importance, studies on the interaction between fibroblasts and T cells as therapeutic approaches have been constantly investigated. Nevertheless, further research is needed to understand the interaction between fibroblasts and T cells, which might improve the therapeutic advantages for various diseases.

## Author contributions

BL drafted the manuscript, figures. S-HL and KS contributed to study conception and revised the manuscript. All authors contributed to the article and approved the submitted version.
